# Comparative analysis of chromosome numbers and sex chromosome systems in Paraneoptera (Insecta)

**DOI:** 10.3897/CompCytogen.v15.i3.71866

**Published:** 2021-09-27

**Authors:** Valentina G. Kuznetsova, Ilya A. Gavrilov-Zimin, Snejana M. Grozeva, Natalia V. Golub

**Affiliations:** 1 Zoological Institute, Russian Academy of Sciences, Universitetskaya emb. 1, St. Petersburg, 199034, Russia Zoological Institute, Russian Academy of Sciences St. Petersburg Russia; 2 Institute of Biodiversity and Ecosystem Research, Bulgarian Academy of Sciences, Blvd Tsar Osvoboditel 1, Sofia 1000, Bulgaria Institute of Biodiversity and Ecosystem Research, Bulgarian Academy of Sciences Sofia Bulgaria

**Keywords:** Chromosome number variability, holokinetic chromosomes, monocentric chromosomes, rates of chromosome number evolution, sex chromosomes

## Abstract

This article is part (the 4^th^ article) of the themed issue (a monograph) “Aberrant cytogenetic and reproductive patterns in the evolution of Paraneoptera”. The purpose of this article is to consider chromosome structure and evolution, chromosome numbers and sex chromosome systems, which all together constitute the chromosomal basis of reproduction and are essential for reproductive success. We are based on our own observations and literature data available for all major lineages of Paraneoptera including Zoraptera (angel insects), Copeognatha (=Psocoptera; bark lice), Parasita (=Phthiraptera s. str; true lice), Thysanoptera (thrips), Homoptera (scale insects, aphids, jumping plant-lice, whiteflies, and true hoppers), Heteroptera (true bugs), and Coleorrhyncha (moss bugs). Terminology, nomenclature, classification, and the study methods are given in the first paper of the issue (Gavrilov-Zimin et al. 2021).

## Introduction

The structure of chromosomes and mechanisms of their evolution, the number of chromosomes and the chromosomal sex determination constitute the foundation of reproduction and are crucially important for reproductive success.

### Chromosome structure and mechanisms of chromosome evolution in Paraneoptera

Nearly all Paraneoptera insects are characterized by holokinetic chromosomes. The only intriguing exception to this rule is the order Thysanoptera (thrips) in which all so far studied representatives of both suborders, Terebrantia and Tubulifera, were shown to have monocentric chromosomes (Brito et al. 2010). Holokinetic chromosomes are, thus, an additional synapomorphy of Paraneoptera, but without Thysanoptera. Monocentric and holokinetic chromosomes make up two main types of chromosomes among eukaryotes. Chromosome segregation during cell division is known to depend on specific chromosomal regions called centromeres and observed with the light microscope. Accurate chromosome segregation requires each chromosome’s centromere to build a kinetochore, a large and complex protein assemblage that connects chromosomes to microtubules of the mitotic and meiotic spindles to distribute the replicated genome from a mother cell to its daughter cells (Westhorpe and Straight 2013).

Most animal and plant species have monocentric chromosomes with centromeres restricted to a defined chromosomal region, the so-called primary constriction, first described by a German biologist and a founder of cytogenetics, Walther Flemming (Flemming 1882). At mitotic anaphase, spindle microtubules attach to the centromeres (actually kinetochores) and bring chromosomes to the poles with the centromere leading. Holokinetic chromosomes, unlike monocentric chromosomes, do not have a localized centromere, and centromeric determinants are dispersed along their whole or almost whole length. Microtubules become attached along the entire length of holokinetic chromosomes, which, therefore, move as linear bars toward the poles at anaphase (Drinnenberg et al. 2014). Holokinetic chromosomes are often referred to as holocentric chromosomes; accordingly, the phenomenon itself became known as holocentricity. However, holokinetic chromosomes do not have a proper centromere in the sense of a primary constriction connecting sister chromatids, how is the case in monocentric chromosomes. At metaphase, holokinetic chromosomes show sister chromatids separated from each other by a regular distance. The term “holocentric” does not, thus, reflect adequately the morphology and behavior of these unusual chromosomes (Mola and Papeschi 2006; Guerra et al. 2010), a conclusion we agree with.

Holokinetic chromosomes, as distinct from monocentric chromosomes, were recognized as late as the mid-1930s (Schrader 1935), and one of the first groups of insects in which these chromosomes were discovered and studied were paraneopteran insects, namely mealybugs (Pseudococcidae, Homoptera) (Hughes-Schrader and Ris 1941; Hughes-Schrader 1942, 1948; Hughes-Schrader and Schrader 1961). Since then, many original articles, reviews and discussion papers about holokinetic chromosomes have been published and an impressive series of conceptual advances in the topic have accumulated (Nokkala et al. 2004, 2006; Guerra et al. 2010; Melters et al. 2012; Drinnenberg et al. 2014; Zedek and Bureš 2018; Mandrioli and Manicardi 2020; Ruckman et al. 2020; Senaratne et al. 2021). The ancestral insects are believed to be monocentric (Melters et al. 2012; Drinnenberg et al. 2014). Holokinetic chromosomes appear to have evolved independently in multiple eukaryotic lineages by convergent evolution (Melters et al. 2012). Among insects, they are currently known in Odonata (Palaeoptera); Dermaptera (Polyneoptera); Copeognatha [=Psocoptera], Parasita [=Phthiraptera], Homoptera, and Heteroptera (Paraneoptera); Lepidoptera and Trichoptera (Oligoneoptera) (White 1973). More recently, it has been shown that Coleorrhyncha and enigmatic Zoraptera (both from Paraneoptera) also have holokinetic chromosomes (Kuznetsova et al. 2002, 2015; Grozeva et al. 2014). Thus, holokinetic chromosomes occur in every major phylogenetic lineage of Pterygota suggesting that they are likely to have evolved at least four times independently in insect evolution (Drinnenberg et al. 2014; Kuznetsova and Aguin-Pombo 2015).

The transition from monocentric to holokinetic state occurred, apparently, by accident; however, holokinetic state itself is speculated (Zedek and Bureš 2018) to represent a potential advantage in exploiting new habitats and adapting to stressing conditions. On the other hand, number of chiasmata (crossingovers) in holokinetic bivalents is limited to one or two only (Nokkala et al. 2004), what can be an evolutionary disadvantage, once the organism adapts to its new habitat (Zedek and Bureš 2018).

One of the most significant advances is the recent discovery that independent transitions to a holokinetic state in insects were associated with the recurrent loss of a centromeric histone H3 variant (CenH3), which is an essential chromatin component of centromeres in most eukaryotes (Drinnenberg et al. 2014; Senaratne et al. 2021). The question is what happened first: the acquisition of the derived holocentric state or the loss of CenH3. Taking into account that CenH3 loss is only identified in “holokinetic” insect lineages (Drinnenberg et al. 2014), the emergence of the “holokinetic” state is likely to have proceeded and subsequently allowed the loss of CenH3 (Senaratne et al. 2021). This sequence of evolutionary events, i.e. the loss of CenH3 after the establishment of “holokinetic” chromosome structure, is also supported by the fact that some “holokinetic” insects do have CenH3 homologs. These insects could therefore represent an intermediate form, leading to the establishment of the CenH3-deficient state (Senaratne et al. 2021).

### Chromosomal mechanisms of sex determination in Paraneoptera

The vast majority of paraneopteran insects have male heterogametic sex determination system. These insects carry either an XX/XY or an XX/X(0) system. Some species have complex systems with multiple X or (much rarer) multiple Y-chromosomes, which evolve mainly by fissions of the ancestral sex chromosomes. Others possess so-called neo-XY systems that have derived from the X(0) system by the fusion of the X chromosome with an autosome, resulting in a neo-X chromosome, and the homologue is transformed into a neo-Y chromosome (White 1973; Blackman 1995). A number of more complicated sex determination systems are also known in paraneopteran insects. Some insects display haplodiploidy (arrhenotoky) where males are haploid and develop from unfertilized eggs, while females are diploid and develop from fertilized eggs (e.g. thrips, whiteflies and some genera of scale insects). Some insects have paternal genome elimination (PGE) where both sexes develop as diploids, but maleness is determined by inactivation or loss of paternal chromosomes, making males functionally haploid (e.g. true lice, scale insects and whiteflies). Finally, true parthenogenesis exists in some paraneopteran insects occurring either in separate species or on higher taxa level, when female embryos develop from unfertilized diploid eggs (see Vershinina and Kuznetsova 2016).

### Chromosome number evolution in Paraneoptera

One of the basic features of holokinetic chromosomes is that their fragments retain centromere activity and the ability to segregate to the poles, which has been demonstrated in experiments with X-ray irradiation (Hughes-Schrader and Ris 1941; Brown and Nelson-Rees 1961; Hughes-Schrader and Schrader 1961).

Moreover, it has been shown in experiments on the Mediterranean flour moth *Ephestiakuehniella* Zeller, 1879 (Lepidoptera, Pyralidae) that radiation-induced chromosome fragments are regularly inherited by both somatic and germ cells and can be transmitted through more than 50 generations suggesting that they persist as long as their active kinetochore elements are preserved (Marec et al. 2001). Due to this feature, chromosomal rearrangements (tandem fusions, fissions, etc.) can arise and be transmitted to daughter cells at successive cell divisions in holokinetic organisms. In contrast, in organisms with localized centromeres, any cells with changes that result in acentric or dicentric chromosomes will be eliminated as such chromosomes will not segregate normally at mitosis, which will result in a loss of genetic material and probably inviable gametes (Blackman et al. 2000; Escudero et al. 2013). Thus, the presence of centromere is a limiting factor in chromosome evolution within monocentric groups.

Chromosome number is highly variable across insects as a whole (Blackmon et al. 2017). Changes in chromosome number can happen due to different mechanisms (Sylvester et al. 2020). Decrease in number can be a result of chromosome fusions, whereas increase in number can occur due to simple chromosome fissions, the addition or loss of a whole chromosome (aneuploidy as trisomy and monosomy, respectively), or the addition of a whole chromosome set (polyploidy). Theoretically, holokinetic chromosome structure facilitates the successful inheritance of novel fusion chromosomes or fission fragments, and species with holokinetic chromosomes should therefore tolerate structural rearrangements of chromosomes better than species with monocentric chromosomes. Because of this, “holokinetic” taxa should have higher rates of chromosome number evolution compared to “monocentric” taxa (Hill et al. 2019). Indeed, there are a number of great examples of rapid chromosome number evolution in holokinetic organisms, both plants and invertebrate animals. So, a large holokinetic angiosperm genus *Carex* (Linnaeus, 1753) (Cyperaceae) demonstrates an exceptional chromosome number series with nearly continuous range from 2n=12 to 2n=124 and substantial variation of the 2n within many species (see for references Escudero et al. 2013). The holokinetic insect order Lepidoptera provides the most impressive examples of this kind showing the highest variance in chromosome number within a species and between species within a genus, the highest single count and polymorphisms in counts that do not affect fertility in crosses (Hill et al. 2019). So, within a widespread Eurasian butterfly *Leptideasinapis* (Linnaeus, 1758) (Pieridae), the 2n gradually decreases from 106 in Spain to 56 in eastern Kazakhstan (Lukhtanov et al. 2011). The large blue butterfly subgenusAgrodiaetus Hübner, 1822 belonging to the genus *Polyommatus* Latreille, 1804 (Lycaenidae) exhibits unusual interspecific diversity in chromosome number varying from n=10 to n=134 (Kandul et al. 2007). Another blue butterfly species, Polyommatus (Plebicula) atlanticus (Elwes, 1906), displays 2n=ca. 448–452, the highest chromosome number among all the non-polyploid eukaryotic organisms (Bureš and Zedek 2014; Lukhtanov 2015). There are equally outstanding examples among Paraneoptera. The standard karyotype of the human bed bug *Cimexlectularius* Linnaeus, 1758 (Heteroptera, Cimicidae) includes 2n=29(26+X_1_X_2_Y) in males (Ueshima 1979). However, in a number of European populations males have very varied chromosome numbers, 2n=29–37, 40, 42, 47; this variability is explained by an increase of the number of X-chromosomes as a result of intense processes of fragmentation of the original X-chromosomes (Sadílek et al. 2013). An endemic Australian scale insect genus *Apiomorpha* Rübsaamen, 1894 (Coccinea, Eriococcidae) exhibits an extraordinary 48-fold variation in chromosome number (2n=4–192) and extensive chromosomal variation within numerous morphologically defined species (Cook 2000).

Although variations in chromosome number of related species are probably due to both fissions and fusions of holokinetic chromosomes, fusions are suggested to be more common. The point is that a chromosome, be it holokinetic or monocentric, has to display two functional telomeres in order to survive a mitotic cycle. A fusion chromosome always displays functional telomeres originated from the ancestral chromosomes, whereas a fission chromosome has to be able to develop a functional telomere *de novo* (Nokkala et al. 2007). However, the ability of holokinetic fragments to restore telomeres *de novo* by telomerase has been repeatedly confirmed in various insects (e.g. Mohan et al. 2011; Mandrioli et al. 2014; for other examples, see Kuznetsova et al. 2020).

To test whether insects with monocentric and holokinetic chromosomes differ in the amount and rate of chromosomal rearrangements, Ruckman et al. (2020) undertook an extensive analysis of chromosome counts across 22 insect orders. The authors focused, on the one hand, on “monocentric” orders Blattodea, Coleoptera, Diptera, Hymenoptera, Isoptera, Neuroptera, and Phasmatodea and, on the other hand, on “holokinetic” orders Odonata, Hemiptera, and Lepidoptera. To exclude polyploidy as a source of “aberrant” (derived) chromosome numbers, the authors explored two models for the evolution of chromosome number, one of which included both fusion/fission and polyploidy, and the other only fusion/fission events. It is of interest that the analysis covering a total of 4,393 species and 599 genera, and using various approaches detected no significant difference between taxa with different chromosome types suggesting that characteristics other than “holocentricity” and “monocentricity” (e.g. meiotic drive, polyploidy events and population size) can be key to determine rates of chromosome number changes (Ruckman et al. 2020). Although this suggestion was based on extensive analysed material (a large number of species, genera, and orders), it requires more solid confirmation. For example, regarding Paraneoptera, representing one of the largest and most diverse insect lineages, the authors addressed only 1,695 species, while the number of paraneopteran species with known chromosome numbers is several time higher. To expand the possibilities of future analyses, we give below a brief overview of chromosome number variations found in each of the major phylogenetic lineages of Paraneoptera, including Zoraptera, Copeognatha (=Psocoptera), Parasita (=Phthiraptera), Thysanoptera, Homoptera, Heteroptera, and Coleorrhyncha.

## Material and methods

The material for this review study was the representatives of all major phylogenetic lineages of Paraneoptera, including Zoraptera (angel insects), Copeognatha (=Psocoptera; bark lice), Parazita (=Phthiraptera; true lice), Thysanoptera (thrips), Homoptera (scale insects, aphids, jumping plant-lice, whiteflies, and true hoppers), Heteroptera (true bugs), and Coleorrhyncha (moss bugs). In most cases, chromosome analysis was performed using both conventional cytogenetic techniques (Giemsa, C-banding, AgNOR-staining, base-specific fluorochrome banding) and FISH mainly with rDNA and telomeric (TTAGG)*_n_* probes. Methods, terminology, nomenclature, and classification are given in the first paper of this issue (Gavrilov-Zimin et al. 2021).

## Review and discussion

Here, we review and discuss basic data on chromosome numbers and sex chromosome systems separately for each Paraneoptera order or, in some cases (e.g. Homoptera), for suborders within the order.

### Zoraptera

Zoraptera (known as angel insects) are minute, less than 3 mm long, insects of cryptic habits living under bark, in humus, termite nests, etc. This is one of the lesser-known insect lineages in the world in terms of distribution, diversity, mode of life, reproductive biology, genetics, etc. The order currently contains a single genus *Zorotypus* Silvestri, 1913 constituting the family Zorotypidae, with 44 extant and 13 fossil species (Mashimo et al. 2014; Villamizar and González-Montana 2018). The majority of zorapteran species are tropical, with just a few exceptions, e.g. *Zorotypushubbardi* Caudell, 1918 widespread in the United States. *Z.hubbardi* is the first and still the only zorapteran species studied in terms of karyotype (Kuznetsova et al. 2002). Males of this species were shown to have 2n=38(36A+XY) and holokinetic chromosomes. Autosomes can be classified into two size groups, with three larger pairs and six pairs showing an even gradation in size, respectively. X- and Y-chromosomes form a chiasmatic bivalent in meiosis, suggesting that sex chromosome system is of a neo-XY type and could result from an X-autosome fusion in the initial karyotype of 2n=40(38A+X). This is consistent with the fact that in insects the only known chiasmatic sex chromosome system is neo-XY (Blackman 1995). The autosomally derived Y chromosome of *Z.hubbardi* is still homologous with the autosomal part of the neo-X that is evidenced by their synapsis in meiosis. This suggests a relatively recent origin of this sex determining system in *Z.hubbardi* (Kuznetsova et al. 2002).

### Copeognatha

Copeognatha (=Psocoptera; bark lice) are mostly small insects inhabiting terrestrial ecosystems; representatives of certain families are closely related to human dwellings and even considered as pests in storage facilities. Psocoptera are a basal taxon of Paraneoptera (Yoshizawa and Saigusa 2001) comprising 5,941 species in 485 genera and 41 families unequally distributed between the three suborders, Trogiomorpha, Troctomorpha, and Psocomorpha. Trogiomorpha comprise 418 recent species (58 genera, 7 families) and retain most archaic features. Troctomorpha comprise 536 species (86 genera, 9 families) and include highly specialized forms. The largest suborder Psocomorpha, considered as the most evolutionarily advanced group of Copeognatha, comprises 5,028 species (330 genera, 27 families) (Mockford 2018).

To date, 90 bark lice species (about 1.5% of the described ones) from 51 genera and 21 families have been studied cytogenetically. Of these, 80 species (43 genera, 16 families) belong to Psocomorpha (reviewed in Golub and Nokkala 2009; see also Golub et al. 2019), 6 species (5 genera, 3 families) to Trogiomorpha, and 4 species (2 genera, 2 families) to Troctomorpha. Diploid chromosome numbers range from 13/14 (male/female) found in two species of Psocomorpha to 29/30 reported for two species of Trogiomorpha; a sex chromosome system X(0) is characteristic of all bark lice species, with the exception of two species of the family Amphipsocidae (Psocomorpha). *Amphipsocusjaponicus* Enderlein, 1906 and *Kolbiaquisquiliarum* Bertkau, 1882 have a neo-XY system, which evolved due to chromosomal rearrangements involving autosomes and sex chromosomes in an ancestral karyotype with an X(0) system (Golub and Nokkala 2001, 2009). Psocoptera demonstrate a clear mode at 2n=16A+XX/X(0). This chromosome complement is found in nearly 90% of the studied species (71 species, 36 genera, 15 families) and in each of the 3 suborders. It is assumed that the modal karyotype (type or basic) is the ancestral one in the evolution of Copeognatha (Wong and Thornton 1966; Golub 1999; Golub and Nokkala 2009). This assumption is confirmed by the following facts. First, both species with a derived sex chromosome system have 2n=16(14A+neo-XY) in males, indicating that translocation of the X to an autosome has most likely occurred in a male ancestor with 2n=17(16A+X). Second, all known triploid parthenogenetic species, *Valenzuelalabinae* Lienhard, 2006, *V.flafidus* (Stephens, 1836) (Caeciliusidae), *Ectopsocusmeridionalis* Ribaga, 1904 (Ectopsocidae), and *Peripsocussubfasciatus* (Rambur, 1842) (Peripsocidae), have 2n=3x=27(24A+XXX). Their karyotype arose from the putative ancestral one, 2n=18(16A+XX), by adding one more haploid set, n=9(8A+X) (Nokkala and Golub 2002, 2006). The remaining derivative karyotypes most likely evolved through chromosome fusion/fission events leading to an increase or decrease in the number of autosomes in the karyotype. In Trogiomorpha, chromosome fissions seem to have prevailed, whereas in Troctomorpha and Psocomorpha, on the contrary, the fusion processes predominated. However, there is still very little data to confirm this hypothesis.

Chromosomes of Copeognatha are comparatively small and of similar size being, therefore, hard to distinguish from each other in the karyotype when the standard techniques of chromosome staining are used. The applying of banding techniques is scarce in this group. Only three species, all from the suborder Psocomorpha, *Psococerastisgibbosa* (Sulzer, 1766), *Blasteconspurcata* (Rambur, 1842), and *Amphipsocusjaponicus*, were studied using C-banding, silver impregnation and sequence-specific fluorochromes CMA_3_ and DAPI (Golub and Nokkala 2001; Golub et al. 2004). It was shown that nucleolus organizer regions (NORs) are located differently in these species: on an autosomal bivalent, on the X-chromosome, and on the neo-XY bivalent, respectively. It was also shown that these species have a small amount of C-heterochromatin, which localizes as a tiny blocks in terminal regions of chromosomes and consists of AT-rich DNA, except for the NOR regions, which are both AT- and GC-rich, just like the X-chromosomes. Some minor differences in the above characters were observed between species and between different chromosomes of a particular species. It was repeatedly shown, that fluorescence in situ hybridization (FISH) helps identifying individual chromosomes and tracing, thus, their behavior in meiosis of “holokinetic” insects (Panzera et al. 2012, 2015; Maryańska-Nadachowska et al. 2013, 2018; Mandrioli et al. 2014; Kuznetsova et al. 2015; Anjos et al. 2016; Golub et al. 2017; Salanitro et al. 2017; Grozeva et al. 2019). Some higher insect taxa were shown to differ in the presence/absence of the insect-type telomere motif (TTAGG)*_n_* (reviewed in Kuznetsova et al. 2020). Within Copeognatha, several Psocomorpha species were studied using FISH (Frydrychová et al. 2004; Golub et al. 2019). In each of these species, 18S rDNA-FISH revealed two large clusters of the rRNA genes located on a medium-sized bivalent, and (TTAGG)_n_-FISH revealed signals at the telomeres of their chromosomes. These data suggest that Copeognatha, at least in the suborder Psocomorpha, display the telomere motif (TTAGG)*_n_* considered ancestral for the class Insecta in general (Frydrychová et al. 2004; Kuznetsova et al. 2020).

### Parasita

Parasita (=Phthiraptera; true lice), the closest relatives of Copeognatha, include obligate ectoparasites of birds and mammals. The order is divided into Mallophaga (Amblycera + Ischnocera) and Rhynchophthirina known as chewing or biting lice, respectively, and Siphunculata (=Anoplura) known as sucking lice (Smith et al. 2011; Kluge 2020). There are about 5,000 known species, of which 550 species (50 genera, 15 families) belong to Siphunculata; 1,341 species (76 genera, 7 families) to Amblycera; 3,120 species (130 genera, 3 families) to Ischnocera; and only 3 species (genus *Haematomyzus* Piaget, 1869, Haematomyzidae) to Rhynchophthirina (Price et al. 2003; Durden 2019). Chromosomal data, although extremely scarce and fragmentary, are presently known for each of the suborders, with the exception of Rhynchophthirina. Of these, 2 species (2 genera, 2 families) belong to Amblycera, 4 species (3 genera, 2 families) to Ischnocera, and 7 species (5 genera, 5 families) to Siphunculata (Tombesi et al. 1999; Golub and Nokkala 2004; Bressa et al. 2015; for other reference see Tombesi and Papeschi 1993). Surprisingly, among as few as 13 studied species, as many as 7 different chromosome counts were found. These latter vary from 2n=4 to 2n=24, and sex chromosomes were not distinguished in any of the studied species. It is impossible now to establish modal numbers (type or basic numbers) either for Parasita as a whole or for separate taxa within the order. The lowest count is found in Amblycera (*Gyropusovalis* Burmeister, 1838), and the highest count is found in Ischnocera (*Chelopistesmeleagridis* (Linnaeus, 1758) as *Gonioidesstylifer* Nitzsch, 1818).

C-banding experiments failed to reveal constitutive heterochromatin in karyotypes of human head- and-body lice, *Pediculushumanuscapitis* De Geer, 1778 and *Pediculushumanushumanus* Linnaeus, 1758 (Pediculidae, Siphunculata) (Bressa et al. 2015). The genomic study of *P.h.humanus* showed its telomeric DNA to consist of the TTAGG repeats (Kirkness et al. 2010). Using a genotyping approach, Filia et al. (2017) have established that both *P.h.capitis* and *P.h.humanus* reproduce through paternal genome elimination (PGE), an unusual genetic system when males transmit only their maternally derived chromosomes.

### Thysanoptera

Thysanoptera (thrips, also known as thunder flies) encompass minute insects, which are usually only a few mm in length. Most thrips feed on fungi, flowers and leaves of green plants, less often on mosses and detritus. Thrips are distributed worldwide being more abundant in tropical, subtropical, and temperate regions. Thysanoptera are a monophyletic group subdividing into the two suborders, Terebrantia with about 2,400 described species in 8 families, and Tubulifera with about 3,500 species in a single family, the Phlaeothripidae. The majority of known thrips species are placed in the two largest families, the Phlaeothripidae (Tubulifera) and Thripidae (Terebrantia) (Mound and Minaei 2007). Most studied Thysanoptera species possess a haplo-diploid reproductive mode and reproduce via arrhenotoky (Nault et al. 2006) where unfertilized eggs develop parthenogenetically into males, which are always haploid, and fertilized eggs develop into females, which are always diploid; so males only transmit their maternal genome to the offspring (see Vershinina and Kuznetsova 2016). A number of thrips are known to be obligatory thelytokous (Nguyen et al. 2015). Moreover, three reproductive modes, thelytoky, arrhenotoky, and even deuterotoky, were documented in *Thripstabaci* Lindeman, 1889 populations collected from onion fields in New York (Nault et al. 2006).

The degree of cytogenetic knowledge of thrips is negligible. Risler and Kempter (1961) compiled all chromosome numbers known for Thysanoptera (10 species in total) studied by the beginning of 60s of the last century. Another 7 species originating from Northeast Brazil were karyotyped by Brito et al. (2010). Most of what is known concerning the chromosomes of thrips is due this latest publication. According to Brito et al. (2010), karyotypes are currently known for 17 species (about 0.3% of the described ones) of which 6 species in 4 genera are from the Phlaeothripidae and 11 species in 9 genera are from the Thripidae. It was unexpected to find out that thrips, unlike all other Paraneoptera insects, have monocentric chromosomes. The lowest and the highest haploid numbers of chromosomes in male thrips are 10 (i.e., 2n=20) and approximately 50–53 (i.e., 2n=100–106), respectively. The lowest count was found in both Tubulifera (*Haplothripstritici* (Kurdjumov, 1912)) and Terebrantia (*Taeniothripssimplex* Morison, 1930), and the highest count is found in Terebrantia, in *Aptinothripsrutua* (Prussard-Radulesco, 1930). Chromosome numbers represent a nearly continuous range in each of the suborders, varying in Tubulifera from n=10 to n=15, with intermediate values 12, 13, 15 and in Terebrantia from n=10 to n=50–53, with intermediate values 14, 15, 16, 17, 18, 20, 21. In *Heliothripshaemorrhoidalis* (Bouche, 1833) and *Taeniothripsinconsequens* (Uzel, 1895), both from the Thripidae, different chromosome numbers were reported by different authors (n=16, 21 and 26/28, and n=16 and 18/20, respectively); however, no information other than chromosome counts is given in the original publications (see for references Risler and Kempter 1961 and also Brito et al. 2010). Besides, two “cytotypes” were found by Brito et al. (2010) in *Gynaikothripsuzeli* (Zimmermann, 1900) (Phlaeothripidae) originating from two isolated localities, one with n=13 and karyotype formula of n=4M+8SM+1A, and the other with n=15 and karyotype formula of n=4M+10SM+1A. Brito et al. (2010) also constructed karyotype formulas for another four species and for each of the *G.uzeli* “cytotypes”. A great variation in both number and morphology of chromosomes was revealed at family, genus and species levels, with usually no clear correlations between changes in chromosome numbers and karyotype structure. The exception seems to be the two studied representatives of the genus *Gynaikothrips* Zimmermann, 1900, *G.uzeli* (with its two cytotypes) and *G.ficorum* Marchal, 1908. The species status of *G.ficorum* remains uncertain for many years. Mound et al. (2016) have suggested that *G.ficorum* is probably a form of *G.uzeli*, and morphological differences between different populations of these thrips can be due to different hosts and latitude. According to Brito et al. (2010), differences in chromosome number between *G.uzeli* (the cytotype A with n=13) and *G.ficorum* (n=15) as well as differences in chromosome morphology between *G.uzeli* (the cytotype B with n=4M+10SM+1A) and *G.ficorum* (n=4M+7SM+6T) indicate that both *G.uzeli* and *G.ficorum* are distinct species and that pericentric inversions led to the differentiation of their karyotypes. It should be noted, however, that both hypotheses, especially the last one, need further research and more convincing chromosomal and also molecular confirmations.

Unfortunately, based on the available data, it is currently impossible to speculate on mechanisms underlying chromosome number diversity in Thysanoptera. It can be assumed that some species of thrips are polyploids. As mentioned above, thelytokous reproduction occurs in some species, including the greenhouse thrips, *Heliothripshaemorrhoidalisis*. This species was suggested to reproduce by automixis through fusion between the second polar body nucleus and the egg nucleus, so called terminal fusion (Bournier 1956). This conclusion was made on the basis that chromosome number was reduced during oogenesis from 2n=42 to 2n=21, followed by the re-establishment of 42 chromosomes. More recently, the analysis of genetic diversity at two nuclear loci has indicated that *H.haemorrhoidalis* may be polyploid (and potentially hexaploid), which does warrant future cytogenetic investigation (Nguyen et al. 2015).

Sex chromosomes were not detected in any species of thrips (Brito et al. 2010), and the cytogenetic mechanisms involved in sex determination in these insects are not yet fully understood. We can assume that they have a chromosomal single-locus sex determination, as is the case with haplodiploid Hymenoptera where sex determination involves no heteromorphic sex chromosomes and sex is determined by a single locus (heterozygotes at the sex locus develop into females and hemizygotes develop into males) (Wilgenburg et al. 2006). The presence of the (TTAGG)*_n_* telomere motif was detected in chromosomes of *Parthenothripsdracaenae* (Heeger, 1854) (Frydrychová et al. 2004).

### Coccinea

Coccinea (scale insects) are sap-sucking phytophagous insects, among which there are many pests of agricultural and ornamental plants, as well as producers of natural dyes, lacquers and waxes. Scale insects represent a moderate-sized group of sternorrhynchous Homoptera comprising more than 8,000 species in the world fauna; they are subdivided into 19–36 recent families, depending on the taxonomic and phylogenetic views of different authors. Here, we follow the traditional system of 19 “large” families, without dividing Margarodidae, Pseudococcidae, Eriococcidae, and Asterolecaniidae into minute “families” with overlapping characters (see for details Danzig 1980; Gavrilov-Zimin 2018a). Scale insects have a wide range of chromosome numbers and also unique variety of sex determination mechanisms and mode of reproduction, including hermaphroditism, paternal genome heterochromatinization (differential inactivation) and elimination (PGH and PGE, respectively), different types of haplodiploidy (both usual haplodiploidy and the so-called Lecanoid and Diaspidoid systems with some variations), various modes of parthenogenesis, etc. (Hughes-Schrader 1948; Nur 1980; Gavrilov 2007; Gavrilov-Zimin et al. 2015). As mentioned at the beginning of this paper, mealybugs (Pseudococcidae) were one of the first groups in which holokinetic chromosomes were discovered as a phenomen and studied (Hughes-Schrader and Ris 1941; Hughes-Schrader 1942, 1948; Hughes-Schrader and Schrader 1961). Scale insects, especially some mealybugs (*Planococcus* spp.), have long been used as a model system to study the mechanisms underlying resistance of holokinetic chromosomes to ionizing radiation as well as the phenomenon of genome stability in holokinetic organisms (Hughes-Schrader and Ris 1941; Brown and Nelson-Rees 1961; Hughes-Schrader and Schrader 1961; Mohan et al. 2012).

Chromosome numbers have been reported for 506 species of scale insects belonging to 15 families (reviewed by Gavrilov 2007 and Gavrilov-Zimin et al. 2015; see also Gavrilov-Zimin 2016, 2017, 2018b, 2020), thus comprising about 6% of the total number of coccid species described to date. The lowest chromosome number, 2n=4, is found in 21 species of the tribe Iceryini (Margarodidae) and in several species of the endemic Australian gall-inducing felt-scale genus *Apiomorpha* (Eriococcidae), while the highest number, 2n≈192, is found in *Apiomorphamacqueeni* Froggatt, 1929 (Hughes-Schrader 1948; Cook 2000, 2001; see for review Gavrilov 2007). Three values, 2n=8, 10, and 18, can be suggested today as modal ones for Coccinea as a whole. They prevail in large and comparatively better studied families Pseudococcidae s.l. (138 species in 50 genera), Eriococcidae s.l. (98 species in 19 genera), Diaspididae (141 species in 68 genera), Coccidae (56 species in 31 genera), and Margarodidae s.l. (39 species in 21 genera). Moreover, most studied species of the small families Dactylopiidae and Phoenicococcidae s.l. have 2n=10, and the only species of the ancient monotypic family Phenacoleachiidae, *Phenacoleachiazealandica* (Maskell, 1891), has 2n=8. All other families are very poorly studied (Ortheziidae, Xenococcidae, Kermesidae, Aclerdidae, Conchaspididae, Asterolecaniidae s.l., and Kerriidae) or not studied at all (small families Carayonemidae, Stictococcidae, Micrococcidae, and Beesoniidae) (see Gavrilov 2007; Gavrilov-Zimin et al. 2015).

In some cases, an increase in the number of chromosomes is a result of polyploidy or the presence of B-chromosomes. Both triploid and tetraploid forms were described by Nur (1979) in the soft scale (Coccidae) species *Physokermeshemicryphus* (Dalman, 1826) and *Saissetiacoffeae* (Walker, 1852). We can assume that polyploidy actually occurs in scale insects more often what is indirectly indicated by that some congeneric species of soft scales, felt scales, and mealybugs differ from each other in chromosome number three or four times (Gavrilov 2007). The presence of some chromosomes, additional to the standard chromosome number and suggested to be B-chromosomes, has been confirmed in many publications (Gavrilov 2007). However, only in some species, e.g. *Pseudococcusviburni* (Signoret, 1875) (Pseudoccocidae), B-chromosomes were studied in more details (Nechaeva et al. 2004). In this species, B-chromosomes were found to be large (these chromosomes are usually small although exceptions are known, e.g. Maryańska-Nadachowska 1999) and completely heterochromatic (after C-banding); they varied in number from zero to 2 within an individual, with a single B- chromosome being most frequent.

The number of chromosomes is quite often stable or slightly variable within genera and within many higher rank taxa of scale insects (see Gavrilov 2007; Gavrilov-Zimin et al. 2015). Here are examples coming from some comparatively better studied groups. The mealybug genera *Pseudococcus* Westwood, 1840 and *Phenacoccus* Cockerell 1893 (Pseudococcidae) are characterized by a karyotype with 2n=10, which was found in 13 species (of the 15 studied ones) and in 19 species (of the 20 studied ones), respectively. The felt scale genus *Eriococcus* Targioni Tozzetti, 1868 (Eriococcidae) was found to have 2n=18 in 13 of the 18 studied species. In the family Diaspididae, which is most species-rich (2,650 species in 418 genera) and the best cytogenetically studied (141 species in 68 genera), the 2n varies from 6 to 18 but the overwhelming majority of species have 2n=8. However, there are exceptions. Some scale insect genera demonstrate a significant or even extraordinary variation of chromosome number; moreover, different diploid numbers can be found in the same nominal species. Most impressive example of such variation is the aforementioned feltscale genus *Apiomorpha*, where 42 diploid counts, ranging from 2n=4 to 2n≈192, have been found in 47 studied species, including undescribed ones. Moreover, extensive chromosomal variation was observed within many morphologically defined species of *Apiomorpha*, suggesting the involvement of chromosomal changes in the divergence of this lineage and in the generation of cryptic species (Cook 2000, 2001; Mills and Cook 2010).

Scale insects are characterized by a huge variety of mechanisms of sex determination (see for references Nur 1980; Gavrilov-Zimin and Kuznetsova 2007; Gavrilov 2007; Gavrilov-Zimin et al. 2015). Sex chromosomes have been identified in only a small number of species. The XX/X(0) system was reported for some primitive taxa, including genera of the most ancient families Margarodidae and Ortheziidae (archaeococcids) (see for references Gavrilov-Zimin 2018a) and was assumed to be ancestral for Coccinea in general (Nur 1980; Gavrilov-Zimin and Danzig 2012). The same system was also identified in several studied species of the genus *Puto* Signoret, 1875 from the family Pseudococcidae (neococcids). There are known species with multiple sex chromosomes that have arisen presumably because of fissions (fragmentations) of the original X-chromosomes. *Matsucoccusgallicolus* Morrison, 1939 (Margarodidae) is a unique species with 12Xs in females and, thus, 6Xs in males (Hughes-Schrader 1948), the number being probably the highest among both Paraneoptera and Insecta in general. Although even higher numbers of X-chromosomes (up to 21) were reported for a bed bug species *Cimexlectularius* Linnaeus, 1758 (Heteroptera) (Sadílek et al. 2013), they all exist in the form of polymorphism.

Currently, there are no confirmed cases of the XY system in Coccinea. Two species, *Praelongortheziapraelonga* (Douglas, 1891) with 2n=16 (Ortheziidae) and the Australian felt scale *Lachnodiuseucalypti* (Maskell, 1892) with 2n=18 (Eriococcidae), were shown to have the same number of chromosomes in both males and females. It has been reported (see for references Gavrilov 2007) that these species do not have the PGH/PGE systems, what could explain the observed phenomenon, and that their males have no morphologically distinguishable (heteromorphic) pair of chromosomes, which would unambiguously assume an XY system. Such an uncertain situation is usually called 2n-2n in scale insects. However, it is known that sex chromosomes can be homomorphic, with little or no divergence in size, and such examples are often found in insects, including those of Paraneoptera (e.g. Rebagliati and Mola 2010; Golub et al. 2015). It should also be added that the degree of divergence is not necessarily associated with sex chromosome age, with examples of young heteromorphic systems and, on the contrary, old homomorphic systems (see Furman et al. 2020 for references). To get more information on what kind of sex determination system these species actually have, we need in chromosomal and molecular markers making it possible to identify sex chromosomes in the genome. Molecular methods based on genome coverage from next generation sequencing data, which are commonly used to distinguish sex chromosomes from autosomes and X and Y chromosomes from each other, have never been used in Coccinea. This approach exploits the difference in sex chromosome ploidy between males and females with XX/XY systems considering that X-linked genes show half the number of genomic reads in males compared to females, and that Y-linked reads are absent in females (Palmer et al. 2019). New cytogenetic methods are still very rarely used in the cytogenetics of scale insects. Nechaeva et al. (2004) used several chromosome banding techniques to study *Pseudococcusviburni*. This species is known to have a Lecanoid sex determination system, which is characterized by heterochromatinization and genetic inactivation of one haploid set of chromosomes in male embryos; however, genes for *rRNA* were shown to remain active in the heterochromatic haploid set of male embryos. Mohan et al. (2011) detected the (TTAGG)*_n_* telomere motif in chromosomes of *Planococcuslilacinus* (Cockerell, 1905). Moreover, these authors also detected telomerase activity at the sites of chromosome breaks initiated by radiation in this species, which implies the possibility of *de novo* telomere formation at the ends of the fragmented chromosomes.

### Aphidinea

Aphidinea (aphids) are a moderate-sized group of sternorrhynchous Homoptera, with approximately 5,000 described species distributed mainly throughout the temperate regions of the globe (Favret and Eades 2009). Aphidinea are considered as a sister taxon to Coccinea (e.g. Shcherbakov 2007) and are sometimes united with it in the higher taxon Aphidococca (Kluge 2010; Gavrilov-Zimin et al. 2015). The close relationship of these groups is well supported by numerous morphological, anatomical, embryological, cytogenetic, physiological and other characters. Aphids are small plant sucking insects, very important as agricultural pests and active vectors of crop viruses. They typically exhibit cyclical parthenogenesis, alternating a single annual (sometimes biennial) bisexual generation with several (or numerous) unisexual (all-female) generations reproducing by apomictic parthenogenesis. The bisexual generation may be lost secondarily, so that reproduction is then exclusively by thelytoky (Blackman et al. 2000).

Opinions differ as to the higher classification within Aphidinea, in particular regarding the number of accepted families and their relationships. In the well-known taxonomic catalogue of aphids (Remaudière and Remaudière 1997), all recent “true aphids” are placed in the only family, Aphididae. However, different authors accept 6 to 13 true aphid families, in addition to the families of “not true aphids”, Adelgidae and Phylloxeridae (Börner 1952; Shaposhnikov 1964; Heie 1987; Heie and Wegierek 2009a, 2009b). Along with two latter families, we accept 12 more recent families of Aphidinea; by now, chromosome numbers have been reported for 1,113 species belonging to all but one (Tamaliidae) families, which is about 22% of the total number of described aphid species (reviewed in Gavrilov-Zimin et al. 2015). The smallest 2n currently known in aphids is 4 being reported for three species of the family Aphididae, *Gypsoaphisoestlundi* Hottes, 1930, *Myzaphisrosarum* (Kaltenbach, 1843), and *Amphorophoratuberculata* Brown et Blackman, 1985. This chromosome number was also reported for a population of *Chaitophoruseucomelas* Koch, 1854 (Chaitophoridae) from Peru; however, several other populations of this species sampled from Israel, Great Britain, and South Africa were found to have high chromosome numbers, 36 or 40 (see for references Gavrilov-Zimin et al. 2015). The highest count, 2n=72, is found in *Amphorophorasensoriata* Mason, 1923 (Blackman 1980).

Like the aforementioned scale insect genus *Apiomorpha* showing the entire chromosome number range known in Coccinea, the aphid genus *Amphorophora* Buckton, 1876 is unique among Aphidinea demonstrating the entire range of chromosome numbers, from 4 to 72, known in aphids (Blackman 1980, 1985). This genus was shown to have 10 different chromosome counts (4, 10, 12, 14, 18, 20, 30, 40, 48, and 72) in 19 studied species and subspecies (see Gavrilov-Zimin et al. 2015). Chromosome numbers vary, although within narrower limits, in some other genera of Aphidinea, e.g. *Phylloxera* Boyer de Fonscolombe, 1834 (6–22), *Glyphina* Koch, 1856 (8–55), *Forda* von Heyden, 1837 (18–30), *Tetraneura* Hartig, 1841 (10–26), *Cinara* Curtis, 1835 (10–22), *Lachnus* Burmeister, 1835 (8–38), *Euceraphis* Walker, 1870 (8–22), *Chaitophorus* Koch, 1854 (4–40), and *Trama* von Heyden, 1837 (8–23) (see Gavrilov-Zimin et al. 2015). Almost permanently parthenogenetic genus *Trama* shows great interspecific and intraspecific diversity of structurally heterozygous karyotypes (Blackman et al. 2000). Only in a few collecting sites of southern England, chromosome number was found to vary from 14 to 23 in *T.troglodytes* von Heyden, 1837, from 9 to 12 in *T.caudata* del Guercio, 1909, and from 10 to 14 in *T.maritima* (Eastop, 1953). It suggests a high rate of karyotype evolution in the above species. The observed karyotype variability appeared to have no association with host plant. This is so even with the most polyphagous species, *T.troglodytes*, which evidences that no host race or biotype formation is occurring or even may occur in the absence of bisexual reproduction (Blackman et al. 2000). This hypothesis is consistent with DNA sequence data evidencing that specimens of *T.troglodytes* sampled from different plants have the same (or very similar) mitochondrial DNA haplotype (Normark 1999). An association between chromosome number and host plant has been described in the corn leaf aphid *Rhopalosiphummaidis* (Fitch, 1856), which has 2n=10 on barley in the northern hemisphere but 2n=8 on maize, sorghum, and Johnson grass (*Sorghumhalepense* (Linnaeus, 1753) in all parts of the world (Brown and Blackman 1988), and in species of the genus *Sitobion* Mordvilko, 1914, which show 2n=12 on ferns while 2n=18 on grasses (Hales et al. 1997).

The karyotypes of some *Trama* species were found to include a variable number of small mainly heterochromatic chromosomal elements. It is of interest that intraspecific changes in 2n mostly involved these heterochromatic elements but not the euchromatic chromosomes, which remain relatively stable in both number and size (Blackman et al. 2000). In bisexual aphid species that display an XX/X(0) sex chromosome composition, X-chromosomes are known to have much heterochromatin and also carry *rRNA* genes (Blackman 1985; Mandrioli et al. 1999; Criniti et al. 2009). The rDNA-FISH experiments carried out on different *Trama* species, showed one to six hybridization signals on heterochromatic elements suggesting that they could have originated from the redundant X chromatin and most likely represent B-chromosomes (Blackman et al. 2020). In species of the genus *Euceraphis* and some related genera, one or more B-chromosomes, also presumably of X chromosomal origin, have also been identified or at least suggested (Blackman 1988). Despite the above cases of chromosome number variability, most aphid genera have a remarkably stable number of chromosomes. For example, in the large genus *Dysaphis* Börner, 1931, all of the 31 studied species were shown to have 2n=12; in the species-rich genus *Aphis* Linnaeus, 1758, the absolute majority of nearly 100 studied species have 2n=8 (for other examples, see Gavrilov-Zimin et al. 2015).

Karyotypes including 2n=8, 10 and 12 can be considered today as modal karyotypes for Aphidinea as a whole. These numbers clearly prevail in the largest (3,035 species in 273 genera) and the best cytogenetically studied (601 species in 119 genera) family Aphididae. These numbers or, at least, some of them are found and prevail in other relatively better studied families, including Drepanosiphidae (141 studied species in 48 genera; 2n=6–48 with the numbers 8, 4, and 18 being, accordingly, most common), Eriosomatidae (86 species in 28 genera; 2n=6–38 with 10, 12, 20 being most common), and Lachnidae (72 species in 11 genera; 2n=6–60 with 10, 12, 14 being most common). Finally, in the family Hormaphididae (with 25 studied species in 9 genera; 2n=8–50), 2n=12 was more common than others. All other families are poorly studied without giving the opportunity to identify modal values.

Like some primitive scale insects, aphids have an XX/X(0) sex determination. The transition between parthenogenetic and bisexual reproduction in the complicated aphid life cycle involves a number of peculiar cytogenetic processes still not studied and understood in necessary details. For example, in order for cyclical parthenogenesis to occur, all the progeny developing from fertilized eggs has to be XX female, whereas all sperm must have only one X-chromosome. This is brought about by the elimination of one of the two X-chromosomes during the single maturation division of the parthenogenetic egg what happens once a year (Orlando 1974; Blackman and Hales 1986; Blackman 1987; Jaquiéry et al. 2012). On the other hand, the formation of the female-only parthenogenetic progeny from the bisexual population involves the elimination of male gametes that do not carry an X. Thus, the X-chromosome of aphids is transmitted half of the time by males and half of the time by females (see the whole annual cycle of *Acyrthosiphonpisum* (M. Harris, 1776) on Fig. 1 in Jaquiéry et al. 2012). In aphids, sex determination, instead of being achieved by stochastic combination of male and female chromosome sets during fertilization (as it happens with other X(0) organisms), is mediated by endocrine factors in response to environmental stimuli (Manicardi et al. 2015). Such a complicated and unique (possibly within Insecta in general) system highlights a special “Aphidoid type” of sex determination in parallel with such unusual systems as Lecanoid and Diaspidoid known in Coccinea (Gavrilov-Zimin et al. 2015).

**Figure 1.**
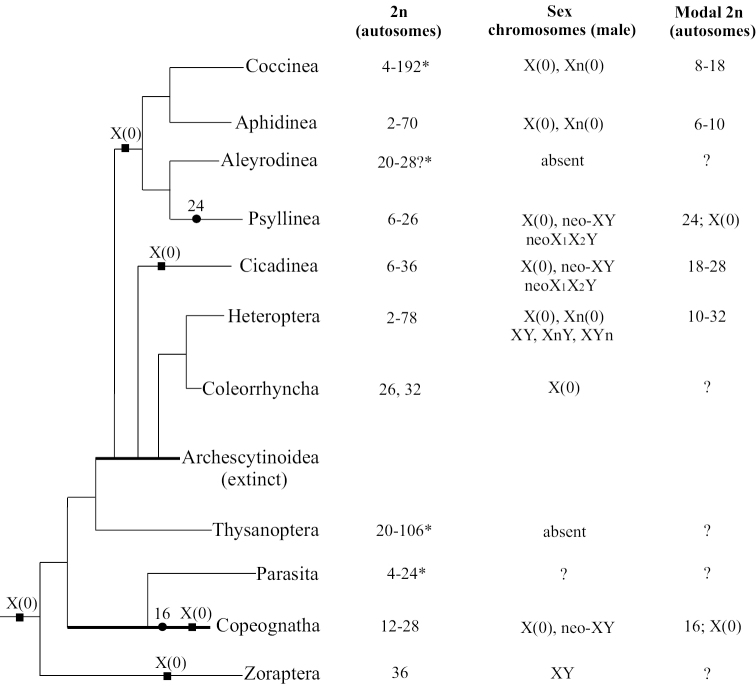
The mapping of diploid autosome numbers, male sex chromosome systems, and both modal and putative ancestral states of these characters onto phylogenetic tree of Paraneoptera. The phylogenetic tree is based on Shcherbakov and Popov (2002), Kluge (2020), and Gavrilov-Zimin (2020a), with modifications. Putative ancestral autosome numbers (2n) are indicated by black solid circles (●); putative ancestral sex chromosome systems are indicated by black solid squares (■).*Total 2n (sex chromosomes not identified) in female.

In addition to the aforementioned *Trama* spp, a number of other aphid species have multiple sex chromosomes originated most likely via X-chromosome fissions, although other mechanisms can also be suggested. Some species of the families Adelgidae and Greenideidae have up to four pairs of X-chromosomes, and some species of the families Phylloxeridae, Eriosomatidae, Lachnidae, and Drepanosiphidae have two pairs of sex chromosomes (see Gavrilov-Zimin et al. 2015). In all above species, despite the presence of numerous sex chromosomes, the sex determination system still remains the same, X_n_X_n_/X_n_(0) (male/female). Hales (1989) described a peculiar X-chromosome constitution in the obligately holocyclic species *Schoutedenialutea* (van der Goot, 1917) (Greenideidae). The female karyotype of this species consists of 4 long chromosomes and 12 short chromosomes. Some embryonic cells, assumed to be male cells, showed 12 short and only 2 long chromosomes suggesting that *S.lutea* has 2n=12A+X_1_X_1_X_2_X_2_/X_1_X_2_ (female/male). This sex chromosome constitution, which is complex in itself, is further complicated by the fusion between sex chromosomes and a pair of autosomes leading to that the sex chromosome constitution of this species turns into an even more complex structure (X_1_+A_1_; X_2_+A_2_; X_1_; X_2_). The latter might be expected to persist only if it conferred some powerful adaptive advantage to the species; however, speculations on possible explanations were argued to be premature in the absence of information on male meiosis in *S.lutea* (Hales 1989).

Despite the large number of karyotyped species, there is not much information yet about the chromatin structure and organization of aphid chromosomes. However, the information that is available is very interesting (e.g. Blackman 1985; Mandrioli et al. 1999, 2014; Blackman et al. 2000; Criniti et al. 2009; Monti et al. 2011; Manicardi et al. 2015). Genes for *rRNA* (*18S*, *5.8S*, *28S*), known to be located on both autosomes and sex chromosomes in other holokinetic groups (see elsewhere), are located (except for a few cases) in aphids exclusively on X chromosomes and mostly correspond to the C-positive heterochromatic areas at one of their ends (see Manicardi et al. 2015). Such a conserved position of C-blocks and *rRNA* genes is probably due to the fact that they are involved in the association of X chromosomes to each other and in the delay in their separation from each other during the complicated processes of transition between parthenogenetic and sexual reproduction in aphids (Orlando 1974; Blackman and Hales 1986). At least four aphid species were shown to have the insect-type motif of telomere (TTAGG)*_n_* (see for references Vershinina and Kuznetsova 2016); however, in the Russian wheat aphid, *Diuraphisnoxia* (Kurdjumov, 1913), this telomeric sequence was not identified (Novotna et al. 2011). An interesting finding made on the TTAGG-positive *Myzuspersicae* (Sulzer, 1776) is that its telomerase is capable of producing telomeres *de novo* to stabilize chromosome fragments, if they arise (Mandrioli et al. 2014 and references therein).

### Aleyrodinea

Aleyrodinea (whiteflies) represent a small suborder of sternorrhynchous Homoptera with a single family Aleyrodidae that includes about 1,600 described species in the world fauna (Ouvrard and Martin 2018). Whiteflies are small (1–3 mm in body length) sap-sucking phytophagous insects; some species are serious pests of various crops and ornamentals while others are capable of vectoring viruses in agricultural crops. Whiteflies are of cytogenetic interest because of their haploid males (White 1973), although the presumption of haplodiploidy for the group in general is based on scant information coming from very few species (Blackman 1995; Blackman and Cahill 1998).

To date, only four whitefly species have been studied cytologically. Male haploid chromosome numbers are known for *Trialeurodesvaporariorum* (Westwood, 1856), n=11, *Aleurotulusnephrolepidis* (Quaintance, 1900), n=13, and for *Aleyrodesproletella* (Linnaeus, 1758), n=13 and/or 14 (Thomsen 1927). Both male (n=10) and female (2n=20) numbers are known for *Bemisiatabaci* (Gennadius, 1889) complex sampled from 4 populations representing either sibling species or host races and/or biotypes (Blackman and Cahill 1998). To test the aforementioned haplodiploidy hypothesis, the last authors made chromosomal preparations from eggs extracted from both unmated and mated females of *B.tabaci*. They observed that in the first case, the laid eggs had 10 chromosomes, whereas in the second case they had either 10 or 20 chromosomes suggesting, thus, that fertilized females of *B.tabaci* are capable to give bisexual offspring including diploid females and haploid males. These observations show that sex determination in whiteflies is more complex than true haplodiploidy. We can assume that *B.tabaci* exhibits haplodiploidy in the form of paternal genome elimination (PGE) as is the case in some Hymenoptera species (Crozier 1975; Bull 1983), some Coccinea (see above) and in a number of other insects (see Vershinina and Kuznetsova 2016; Gokhman and Kuznetsova 2018). More special research is needed to understand sex determination system of whiteflies. Chromosomes of *B.tabaci* lack detectable centromeres being most likely holokinetic as in their relatives within the Homoptera; finally, no detectable differences in chromosome morphology were found between studied populations of *B.tabaci* (Blackman and Cahill 1998). There are data in the literature suggesting that the telomeres of whiteflies, at least in *B.tabaci* and *Trialeurodesvaporariorum*, consist of the canonical insect telomere motif (TTAGG)*_n_* (Frydrychová et al. 2004; Luan et al. 2018). However, the evidence is clearly insufficient. In the first species, the conclusion about the presence of TTAGG repeats was made according to the positive results of Southern hybridization. This technique is however much less reliable than FISH, since it detects repetitive sequences located not only at telomeric sites (Frydrychová et al. 2004). In the second species, the TTAGG repeats were detected in the bacteriocyte genome (insect cells harboring symbiotic bacteria).

### Psyllinea

Psyllinea (jumping plant-lice) form a moderate-sized group of sternorrhynchous Homoptera comprising nearly 4,000 species (in more than 200 genera) described from across every biogeographic region of the world, most of which from tropical and subtropical regions (Burckhardt and Ouvrard 2012; Ouvrard 2020). As with related aphids (Aphidinea), scale insects (Coccinea), and whiteflies (Aleyrodinea), psyllids feed on the phloem of vascular plants but, unlike them, they are generally oligophages restricted to one or a few closely related host plants, particularly at the larval stage. Several species are harmful to their host plants acting as vectors for various plant diseases, as economically important pests in agriculture and forestry, and as potential biocontrol agents for some invasive plants (Burckhardt et al. 2014; Percy et al. 2018). In a recent classification of Psyllinea (Burckhardt and Ouvrard 2012), as many as 8 families have been recognized incuding Aphalaridae, Carsidaridae, Calophyidae, Homotomidae, Liviidae, Phacopteronidae, Psyllidae, and Triozidae. Cytogenetically, approximately 220 species from 55 genera (about 5.3% and 27% of their total number, respectively) have been investigated to date (Maryańska-Nadachowska and Głowacka 2005; Labina et al. 2007; Kuznetsova et al. 2012; Nokkala et al. 2019; for other references, see review of Maryańska-Nadachowska 2002). Karyotypes, mainly in terms of chromosome number and sex chromosome system, are currently known for all families with the exception of the tropical most basal family Phacopteronidae (Cho et al. 2019). Most data concern representatives of the Psyllidae (Spondiliaspidinae, Psyllinae), Aphalaridae (Aphalarinae, Rhinocolinae), and Triozidae, whereas other families have been relatively poorly studied.

With rare exceptions (Labina et al. 2009; Nokkala et al. 2013, 2015, 2019), psyllids reproduce bisexually. The bisexual species have diploid chromosome numbers varying between 7 and 27 in males with a distinct mode at 25, suggesting that this is the ancestral count of the Psyllinea. The most frequent karyotype, 2n=24A+XX/X(0), is found in approximately 80% and 52% of the studied species and genera, respectively, and in each of the studied families being, therefore, considered as evolutionarily ancestral for Psyllinea as a whole (Kuznetsova et al. 1997; Maryańska-Nadachowska 2002). All other karyotypes could have evolved independently as derived characters in different families. The number of autosomes in derived karyotypes is usually lower than the mode. An exception is the karyotype of *Pauropsyllatricheata* Pettey, 1924 (Triozidae) comprising 2n=26A+X(0) and attributing to the fission of one pair of autosomes (Maryańska-Nadachowska and Głowacka 2005). Other few cases of an increase of chromosome number are associated with B-chromosomes and polyploidy (see below).

Based on available data, it can be suggested that the ancestral Psyllinea lineage experienced a series of chromosomal rearrangements, among which chromosome fusions most likely dominated. Rearrangements, other than fusions/fissions, do not alter chromosome number and size and remain unfortunately undistinguishable in chromosome preparations because of the absence of reliable chromosomal markers. In the majority of known cases only a few chromosome fusions have occurred, resulting in insignificant differences in chromosome numbers between related species. There are two impressive exceptions. Within the family Psyllidae, which displays predominantly 2n=25, the Australian subfamily Spondiliaspidinae is characterized by very low chromosome numbers, 2n=7, 9 or 11, found in males of all so far studied species (16 species, 10 genera, tribes Ctenarytainini and Spondyliaspidini) (Maryańska-Nadachowska et al. 2001). These low-numbered karyotypes suggest their common origin, with 2n=10A+XX/X(0) being supposedly an ancestral trait in this group. Data on chromosome numbers suggest an independent reduction of 2n at least once in Ctenarytainini and at least once in Spondiliaspidini. Another group with low chromosome numbers, 2n=11 and 2n=13 (males), is the subfamily Rhinocolinae (Aphalaridae); however, in this group, data are still available only for 4 species (see Maryańska-Nadachowska 2002).

Undoubtedly, the X(0) sex chromosome system is the ancestral one in Psyllinea. Several species have a neo-XY system or a neo-X_1_X_2_Y system. Two species, *Cacopsyllasorbi* (Linnaeus, 1767) and *C.mali* (Schmidberger, 1836), were reported to have derived sex chromosome systems originating from one or several autosome-autosome and X-autosome fusions (Grozeva and Maryańska-Nadachowska 1995; Maryańska-Nadachowska et al. 2018). In *C.sorbi*, different male karyotypes, 2n=24A+X and 2n=20A+neo-XY, were found in various European populations (Suomalainen and Halkka 1963; Grozeva and Maryańska-Nadachowska 1995; Maryańska-Nadachowska and Grozeva 2001; Maryańska-Nadachowska et al. 2018). The neo-XY cytotype was suggested to have originated from 2n=24A+X through at least two fusions, one between two pairs of ancestral autosomes (resulting in 22A+X), and another between one of the fusion chromosomes and the X. In *C.mali*, a fusion supposedly occurred first between an autosomal pair and the X in the progenitor karyotype 2n=24A+X, resulting in 2n=22A+neo-XY; a further fusion occurred between the neo-XY and another autosomal pair resulting in 2n=20A+neo-X_1_X_2_Y (neo-X_1_X_2_Y cytotype) (Grozeva and Maryańska-Nadachowska 1995). Both forms (the cytotypes) coexist within geographically distant populations and are morphologically indistinguishable from each other (Grozeva and Maryańska-Nadachowska 1995; Maryańska-Nadachowska and Grozeva 2001; Nokkala et al. 2004; Maryańska-Nadachowska et al. 2018). These forms are most likely conspecific. A reason for the neo-X_1_X_2_Y system to be present in a polymorphic state (together with the neo-XY system) has been attributed to the high frequency of unbalanced gametes produced during meiosis in X_1_X_2_Y individuals, thus, resulting in the neo-X_1_X_2_Y system being unable to become fixed in a population (Nokkala et al. 2004).

The intraspecific variation in the chromosome number of Psyllinea is sometimes related to the occurrence of B-chromosome (Maryańska-Nadachowska 2002). B-chromosomes are generally considered as extra chromosomes that are often selfish in their transmission and lack the ability to meiotic pairing unlike A chromosomes. The number of B-chromosomes can vary among populations of the same species, among individuals in a population and among cells in an individual (Camacho et al. 2000; Ahmad and Martins 2019). Two psyllid species, *Rhinocolaaceris* Linnaeus, 1758 and *Cacopsyllaperegrina* (Foerster, 1848), have been studied in detail in terms of B chromosome distribution, C-banding pattern and behavior in meiosis. In *R.aceris*, B chromosomes varied in number (from one to three) in geographically distant populations and differed from each other in size, C-banding pattern and alleged origin (Maryańska-Nadachowska 1999). In male meiosis of both *R.aceris* and *C.peregrina*, a B-chromosome and the X chromosome were observed to appear as univalents during prophase I stages, displayed a “touch and go” pairing at metaphase I, and underwent quite regular segregation at anaphase I (Nokkala et al. 2000, 2003). To account for this peculiar behavior, the authors of the works suggested that a B-chromosome was integrated into an achiasmatic segregation mechanism with the X chromosome in a place normally occupied by a Y chromosome in species with achiasmatic XY systems. They hypothesized that Y chromosome may arise from a mitotically stable B-chromosome that was first integrated into an achiasmatic segregation mechanism with the X and, then, became fixed in the karyotype as a Y chromosome. In *R.aceris*, consecutive stages of the conversion of a B-chromosome into a Y chromosome were detected in different populations (Nokkala et al. 2000; Nokkala and Nokkala 2004). It was conjectured that a Y chromosome formed this way was a morphological Y chromosome only and carried no male determining genes (Nokkala et al. 2003). It was suggested that the same mechanism underlined the origin of the achiasmatic Y chromosome in some species of Cicadinea (see Kuznetsova and Aguin-Pombo 2015) and in *Drosophila* (Diptera) (Carvalho et al. 2009), as well as the origin of a W chromosome in Lepidoptera (Fraïsse et al. 2017).

In psyllids, all three known cases of polyploidy (three species) have been detected in the same genus *Cacopsylla* Ossiannilsson, 1970 (family Psyllidae), which is the most diverse genus of Psyllinea, with over 500 known species distributed throughout the Holarctic Region, and spreading into the Oriental Region (Ouvrard et al. 2015). The first of these species, *Cacopsyllamyrtilli* W. Wagner, 1947, is widely distributed throughout the Palaearctic, while its distribution also shows a shift towards the north and/or high altitudes. Females of this species are usually triploid (2n=3x=36A+XXX) and reproduce through apomictic parthenogenesis, while rare diploids also exist. Infrequent, mainly nonfunctional, but also functional males with 2n=24A+X can be found in some populations (Nokkala et al. 2013, 2015). Another species, *Cacopsyllaborealis* Nokkala et Nokkala, 2019, is common and widespread throughout the Palaearctic too. Its distribution range reaches from northern Fennoscandia in the West to Magadan in the East. *C.borealis* is a pentaploid species (2n=5x=60A+XXXXX) with apomictic parthenogenetic reproduction. No males have been recorded in *C.borealis* so far (Nokkala et al. 2019). The third species, *Cacopsyllaledi* (Flor, 1861), is widely distributed throughout Fennoscandia, Central Europe, and Russia; it occasionally forms sympatric populations with *C.borealis*. Its habitats are restricted to the temperate and alpine zones. The species is triploid (2n=3x=36A+XXX) and reproduces through apomictic parthenogenesis, while infrequent functional males (2n=24A+X) can be found in some populations. In such populations with rare males, infrequent diploid females (2n=24A+XX) also exist among the triploids (Nokkala et al. 2015). The above examples demonstrate the unique possibilities of cytogenetic methods for studying the nature of reproduction and the structure of populations of insects.

### Cicadinea

Cicadinea (sometimes referred to as “true hoppers”) are a large group of auchenorrhynchous Homoptera comprising more than 47,000 species distributed worldwide. The true hoppers are generally monophagous or narrowly oligophagous; many species are of economic significance acting as pests of agricultural crops and vectors of plant pathogens, including phytoplasmas, viruses, spiroplasmas, and bacteria. The two major lineages are recognized within the suborder: the infraorder Cicadomorpha comprising four superfamilies, Cicadoidea (cicadas), Cercopoidea (froghoppers and spittlebugs), Membracoidea (leafhoppers and treehoppers), and Myerslopoidea (ground-dwelling leafhoppers), and the infraorder Fulgoromorpha comprising the only superfamily Fulgoroidea (planthoppers) (Bartlett et al. 2018). Cicadomorpha are subdivided into 13 families with approximately 30,000 described species; the Fulgoromorpha is subdivided into about 20 families (depending on the classification followed) with more than 12,000 described species (Dietrich 2002; Cryan 2005; Deitz 2008; Bartlett et al. 2018).

Karyotypes of approximately 850 species (nearly 2% of the total number of species described) belonging to 500 genera of 31 families representing all currently recognized superfamilies of the suborder Cicadinea were studied up to now (reviewed in Kirillova 1986, 1987; Emeljanov and Kirillova 1990, 1992; Kuznetsova and Aguin-Pombo 2015; see also Maryańska-Nadachowska et al. 2016; Anjos et al. 2016, 2018; Anjos 2017; Karagyan et al. 2020). Since the late 1990s, chromosome studies of Cicadinea have been carried out using C- and AgNOR- banding, base-specific fluorochrome-banding, and FISH with a number of DNA probes. The use of these methods and approaches allowed to identify specific regions in chromosomes of Cicadinea and enhanced understanding their chromosome structure (Anjos et al. 2016, 2018; Maryańska-Nadachowska et al. 2016; Anjos 2017; Karagyan et al. 2020; for earlier publications, see Kuznetsova and Aguin-Pombo 2015). In particular, it has been shown that the chromosomes of true hoppers have, like in the majority of other Paraneoptera, the insect (TTAGG)*_n_* motif of telomeres (Karagyan et al. 2020; for other references see Kuznetsova et al. 2020).

According to Kuznetsova and Aguin-Pombo (2015), chromosome numbers in Cicadinea range between 8 and 38 (2n, female); the lowest and the highest numbers are found in Cicadomorpha (Cicadellidae) and Fulgoromorpha (Delphacidae and Dictyopharidae), respectively. Despite the fact that since 2015 new data have appeared, the above series has remained unchanged. Chromosome numbers exceeding 38, when reported, are all related to parthenogenesis (true parthenogenesis referred to as thelytoky or gynogenesis, sometimes to as pseudogamy) and accompanying polyploidy. For example, up to 45 chromosomes have been found in pseudogamous triploid females of *Muellerianellafairmarei* Perris (Booij 1981, 1982) and other planthopper species belonging to the genera *Ribautodelphax* Wagner, 1963 (den Bieman 1988) and *Delphacodes* Fieber, 1866 (den Bieman and de Vrijer 1987). Cicadomorpha and Fulgoromorpha differ both in the limits of variation in chromosome number and in the modal numbers. Within each infraorder, some taxa have more than one modal number and these numbers are characteristically lower in Cicadomorpha than in Fulgoromorpha. In the latter, 2n varies from 20 (*Pentastiridiushodgarti* Distant, 1911 in Cixiidae) to 38 (*Scolops* spp. in Dictyopharidae and *Paraliburniaclypealis* J. Sahlberg, 1871 in Delphacidae), with strongly marked mode at 28 and the second and the third modes at 30 and 26, respectively. In Cicadomorpha, 2n varies from 8 (*Orosius* sp., Cicadellidae) to 32 (*Peuceptyeluscoriaceus* Fallén, 1826, Aphrophoridae). Most species have 2n between 20 and 28, other counts being rare. Specifically, in Cercopoidea 2n varies between 14 and 32, with the mode at 26–28; in Cicadoidea between 12 and 20, with the mode at 20; in Membracoidea between 8 and 28, with the mode at 22 (Emeljanov and Kirillova 1990; Kuznetsova and Aguin-Pombo 2015; Anjos et al. 2018). In Myerslopoidea, the only studied species, *Mapucheachilensis* (Nielson, 1996), has 2n=16+XY. The XY system in this species is most likely of a neo-XY type and indicates the derivative nature of the karyotype, which could be a result of a fusion between the original X and an autosome in the original karyotype of 2n=18+X(0) (Golub et al. 2014).

Some higher taxa of Cicadinea show stable or only slightly variable karyotypes. Quite often, the chromosome number is constant within the genus and even within the family suggesting that fusion/fission events were rare in their evolution. Supporting examples can be found in the review of Kuznetsova and Aguin-Pombo (2015) and in some more recent original publications. Some impressive examples of this sort come from the groups, which have been more fully explored, e.g. the families Dictyopharidae and Issidae (Fulgoroidea), and the cicada genus *Magicicada* Davis, 1925 (Cicadidae, Cicadoidea). The family Dictyopharidae is one of the largest families of planthoppers worldwide, with 720 valid species and 156 valid genera at present (Bourgoin 2016). The family is classified into the two subfamilies, Dictyopharinae and Orgeriinae. The karyotypes are known in 18 species (7 genera) of the first subfamily and in 29 species (17 genera) of the second subfamily. In Dictyopharinae, the tribe Dictyopharini (with about 14% of species and genera studied) is characterized by 2n=28A+X(0) in males. Within Orgeriinae, the most primitive tribe Ranissini (about 20% and 70%, respectively) and one of the most advanced tribes, Orgeriini (about 7% and 20%), show 2n=26A+X(0) in all the species studied. The tribe Almanini (about 20% and 60%) is characterized by 2n=24A+neo-XY. Available data suggest that the ancestral karyotype of the Dictyopharidae included 2n=28A+X(0) and karyotypic transformations in the evolution of the family occurred mainly by fusion of chromosomes (Kuznetsova 1986; Kuznetsova et al. 2009). For the world-wide family Issidae, a lot of new data have been received recently (Maryańska-Nadachowska et al. 2016). The family comprises approximately 1,000 species with around 170 genera classified within the only subfamily Issinae with the three tribes, Issini, Hemisphaeriini and Parahiraciini (Gnezdilov 2013). In general, karyotypes have been studied in 44 (5%) species from 27 (15%) genera covering all the three recognized tribes (Maryańska-Nadachowska et al. 2016 and references therein). Available data suggest that Issidae are a group characterized by a high karyotypic conservatism, which manifests itself primarily in the same karyotype, 2n=26A+XX/X(0), found in all but three studied species. The basic issid karyotype appears also conservative in structure. Every species was shown to have a very large pair of autosomes that also carry rDNA clusters what has been confirmed by both different chromosomal staining techniques (AgNOR banding and DNA-binding fluorochrome CMA_3_) and rDNA-FISH. The newly obtained data support, thus, the hypothesis that the karyotype of 2n=26A+XX/X(0) has the monophyletic origin and represents an ancestral character state for the family Issidae in general (Maryańska-Nadachowska et al. 2006; Kuznetsova et al. 2010). The chromosome number decreased independently at least three times in the evolution of Issidae, and all three reduction events happened in the same tribe Issini. Males of *Latilicamaculipes* (Melichar, 1906) and *Brahmaloka* sp. were shown to have 2n=24A+X, whereas males of *Falcidiuslimbatus* (A. Costa, 1864) were found to have 2n=24A+neo-XY. Both derived karyotypes could have arisen by a single tandem fusion in the ancestral karyotype of 2n=26A+X, the first between two pairs of autosomes resulting in 2n=24A+X, and the second between an autosome and the X resulting in 2n=24A+neo-XY. The genus *Magicicada* (Cicadidae) inhabiting eastern United States and comprising the periodical cicadas remarkable for their 17- or 13-year synchronized life cycles and periodical mass emergence of adults, has 2n=18A+XX/X(0) in all the seven recognized species (Karagyan et al. 2020). Moreover, the same chromosome number seems to be characteristic of the family Cicadidae in general (Kuznetsova and Aguin-Pombo 2015).

The XX/X(0) sex determination is of common occurrence and seems to be an ancestral trait in both Cicadinea (Halkka 1959; Emeljanov and Kirillova 1990, 1992) and their allies (Blackman 1995). Despite evolutionary stability, in some cases the X(0) system has been replaced by an XY system in species within the same genus or even within the same family that are otherwise exclusively X(0), as is the case in the aforementioned family Dicrtyopharidae. Chromosome systems of sex determination evolved via autosome/sex chromosome fusion have been frequently reported in Cicadinea (Kuznetsova and Aguin-Pombo 2015). In a recently formed neo-XY system, the autosomally derived neo-Y chromosome and the autosomal part of the neo-X chromosome remain still homologous, and therefore synapse at prophase I of meiosis. Once a neo-XY system has arisen, it can undergo a further transformation into a multiple X_1_X_2_Y system as a result of the translocation involving the Y chromosome and another pair of autosomes. In representatives of the spittlebug genus *Philaenus* Stål, 1864 (Cercopoidea, Aphrophoridae), which were thoroughly studied using different methods and approaches (AgNOR- and C-banding; fluorochromes CMA_3_ and DAPI; FISH with 18S rDNA and (TTAGG)*_n_* telomeric probes), it was possible to trace almost all successive evolutionary stages of sex chromosome transformations (Maryańska-Nadachowska et al. 2012, 2013). A different, achiasmatic XY system, with a very small Y chromosome, was found in the planthopper species *Limoisemelianovi* Oshanin, 1908 and *L.kikuchii* Kato, 1932 (Fulgoridae) (Kuznetsova 1986, Tian et al. 2004). It seems likely that the Y in such cases has originated from a mitotically stable B-chromosome through a mechanism suggested by Nokkala et al. (2004) and discussed in more detail above, in the psyllid part of this paper.

Theoretically, as mentioned above, fission and fusion of holokinetic chromosomes do not result in unbalanced meiotic products, and so these rearrangements may be preserved through generations and establish variations in chromosome number within populations. Yet, descriptions of chromosomal polymorphisms are quite rare in Cicadinea. We can anticipate that it is due to very few studies at the population level in this group (like in other Paraneoptera). However, some examples of polymorphism for B-chromosomes and for fission/fusion events have been described in natural populations of both leafhoppers and planthoppers (Kuznetsova and Aguin-Pombo 2015). There are also some groups of Cicadinea, in which a wide variety of chromosome numbers occurs suggesting that both fusions and fissions have established themselves during their evolution. For example, in the genus *Eurhadina* Haupt, 1929 (Cicadellidae), a full range from 2n=12 to 2n=20 was reported for only 19 studied species. The cosmopolitan genus *Empoasca* Walsh, 1862 with more than 800 nominal species (Southern and Dietrich 2010) is another group, which seems to show a striking range in chromosome number. In this genus, 12 species examined so far display 2n ranging from 16 to 22. Finally, the aforementioned spittlebug genus *Philaenus* displays 2n=20, 23, and 24 in only 12 studied species. More examples can be found in Kuznetsova and Aguin-Pombo (2015).

### Heteroptera

Heteroptera (true bugs) are a very diverse group in terms of habitats (aquatic, terrestrial and parasitic on vertebrates, including human and birds) and feeding habits (phytophagous, predators, and hematophagous) (Weirauch and Schuh 2011). Several species have received intense focus for economic, medical or scientific reasons (Wang et al. 2019). Heteroptera are the largest order of Paraneoptera with more than 42,000 described species in about 90 families and seven infraorders including Leptopodomorpha, Gerromorpha, Nepomorpha, Pentatomomorpha, Cimicomorpha, Dipsocoromorpha, and Enicocephalomorpha (Štys and Kerzhner 1975; Henry 2017; Weirauch et al. 2019).

The very beginning of cytogenetic studies in Heteroptera dates back to the end of the 19^th^ century when German biologist Hermann Henking (1891) discovered a “peculiar chromatin element” in sperm nuclei of *Pyrrhocorisapterus* Linnaeus, 1758 (Pyrrhocoridae), which he designated in his drawings by “x” (actually a sex chromosome). Since this great discovery, the following key developments have occurred in true bugs cytogenetics. In the period from 1905 to 1912, T.H. Montgomery and E.B. Wilson published a series of papers that actually marked the beginning of true bug cytology (see for references, Ueshima 1979). Hughes-Schrader and Schrader (1961) were the first who established that the chromosomes of true bugs lack centromeres, i.e. they are holokinetic. Ueshima (1979) published a monograph/survey devoted to cytogenetic characteristics of Heteroptera. Some of these characteristics, e.g. presence of a pair of m-chromosomes (see below) and the so-called sex chromosome “post-reduction” in male meiosis when sex chromosomes undergo equational division at anaphase I and reductional division at anaphase II, make true bugs unique among Paraneoptera and even among Insecta in general. The above monograph also contains a comprehensive check-list of chromosome numbers and sex chromosome systems known at that time for all heteropteran infraorders with the exception of Enicocephalomorpha, for which information is lacking to this day. Since Ueshima’s (1979) excellent review, which until now remains the only source of information about chromosome numbers and sex chromosome mechanisms of Heteroptera, and a review by Manna (1984), a large amount of new cytogenetic data on the Heteroptera has been obtained. Papeschi and Bressa (2006) summarized and discussed data accumulated by that time on the basic aspects of true bugs cytogenetics and speculated about mechanisms of karyotype evolution in Heteroptera as a whole. According to this comprehensive review, heavily based on Ueshima’s list, data were available for approximately 1,600 species belonging to 46 true bugs’ families. Although the data set seemed to be impressive, the number of studied species by 2006 was no more than 4.2% of described true bug species. The authors have argued that chromosome numbers in true bugs vary from 2n=4 to 2n=80 but about 70% of the species have 12 to 34 chromosomes, with male diploid number of 14 being the most represented; sex chromosome mechanism is predominantly of the XX/XY type (found in 71.4% of studied species), but other variants such as an XX/X(0) system (14.7%), multiple sex chromosome systems such as X_n_X_n_ /X_n_Y, X_n_X_n_/X_n_(0), and XX/XY_n_ (13.5%), as well as neo-sex chromosomes (0.5%) also occur. The main mechanisms of karyotype evolution in true bugs were argued to be fusions between autosomes, fusions between the X and an autosome, and fissions involved both autosomes and sex chromosomes. It is generally accepted that multiple sex chromosome systems in Heteroptera are the result of sex chromosome fissions (fragmentations) (Ueshima 1979). This is well supported by an example of *Cimexlectularius* that shows from 2 to 20 X-chromosomes in males of different European populations (Sadílek et al. 2013, 2019a, b). It has been hypothesized that tandem duplications in AT-rich regions on the X-chromosome increase the fragility of these regions, which induces fragmentations of the X-chromosome of *C.lectularius* (Sadílek et al. 2019a). In some cases, multiple systems can result from a non-disjunction (Ueshima 1979; Grozeva et al. 2011) or even a duplication of the X-chromosome (Sadílek et al. 2019b).

For a long time, the question of what mechanism, XY or X(0), was the evolutionarily initial in the Heteroptera has been actively debated. Two alternative hypotheses supported by different sources of evidence have been proposed. One of these holds that the XY system has evolved from an X(0) system (Ueshima 1979) while the other assumes that the XY mechanism is plesiomorphic, the existence of the X(0) species being a result of the repeated loss of the Y chromosome during the evolution (Nokkala and Nokkala 1983, 1984; Grozeva and Nokkala 1996; Pal and Vicoso 2015). Last listed authors argue that many true bugs have XY sex chromosomes, with the Y showing a typical reduction in size relative to the X, suggesting extensive loss of gene content on this chromosome. However, the choice between the above hypotheses still remains difficult, at least until data for the basal groups become available. At present, they are completely absent for Enicocephalomorpha and scarce for Dipsocoromorpha, in which 6 species have been studied and both systems have been found, including within the same genus *Pachycoleus* Fieber, 1860 (Grozeva and Nokkala 1996; Kuznetsova et al. 2011).

In karyotypes of many true bug species (within the infraorders Dipsocoromorpha, Nepomorpha, Leptopodomorpha, and Pentatomomorpha), a pair of so-called “m-chromosomes”, has been described (Ueshima 1979; Grozeva and Nokkala 1996; Papeschi and Bressa 2006; Kuznetsova et al. 2011). The origin and significance of these peculiar chromosomes are still obscure. They behave differently from both autosomes and sex chromosomes during male meiosis. As a rule, m-chromosomes are extremely small while in some species they might be of approximately the same size as the autosomes (Grozeva et al. 2009). They are usually asynaptic and achiasmatic throughout early meiotic prophase (Ueshima 1979); however in male *Coreusmarginatus* Linnaeus, 1758 (Pentatomomorpha, Coreidae), m-chromosomes were shown to undergo normal synapsis at pachytene assuming the chiasma formation (Nokkala 1986). The presence or absence of m-chromosomes is a stable character at higher taxonomic levels in the Heteroptera (Ueshima 1979); however, there are exceptions to this rule (Grozeva and Simov 2008). The discovery of m-chromosomes in the families Dipsocoridae and Schizopteridae of the basal infraorder Dipsocoromorpha allows to suggest that m-chromosomes were present in the plesiomorphic karyotype of the Heteroptera in general (Grozeva and Nokkala 1996).

In a fair number of true bug species, the presence of some extra chromosomes in addition to the standard chromosome number has been confirmed (see for references Ueshima 1979; Kuznetsova et al. 2011). Available data point to a significant variability of these supernumeraries in terms of their size, C-heterochromatin amount and distribution, meiotic behavior and impact on segregation of A-chromosomes in the species. In most cases, these extra chromosomes are interpreted as B-chromosomes without any evidence. Poggio et al. (2013) conducted a thorough study of a population of the assassin bug *Zelurusfemoralislongispinis* Lent et Wygodzinsky, 1954 (Reduviidae) polymorphic for the presence/absence of an extra chromosome. In the studied population, males with 2n=22(20A+XY) coexisted with males displaying 2n=23(20A+XY) + extra chromosome. The meiotic behavior of the extra chromosome was highly regular and similar to that of sex chromosomes. Using various cytogenetic approaches combined with a morphometric analysis of chromosomes, the authors concluded that this extra chromosome was an additional X chromosome rather than a B-chromosome (Poggio et al. 2013).

Over the past 15 years, several review papers devoted to individual higher taxa of true bugs have been published, namely, Cimicomorpha (Kuznetsova et al. 2011), Gerromorpha (Fairbairn et al. 2016), and Pentatomomorpha (Souza-Firmino et al. 2020). Besides, a great number of research papers have been published too (e.g. Panzera et al. 2010, 2012; Kuznetsova et al. 2012; Bardella et al. 2012, 2013, 2014; Chirino et al. 2013, 2017; Sadílek et al. 2013, 2019a, 2019b, 2020; Chirino and Bressa 2014; Pita et al. 2014, 2016; Kaur and Gaba 2015; Stoianova et al. 2015; Golub et al. 2016, 2017, 2018; Angus et al. 2017; Gallo et al. 2017; Grozeva et al. 2019; for other references see aforementioned reviews). As a result, the number of karyotyped species increased significantly and knowledge about karyotypes and their evolution in true bugs was expanded.

According to our rough estimates, since the last review (Papeschi and Bressa 2006) the number of cytologically studied true bug species has increased by almost 300 and, thus, reached approximately 1,900 (5% of described species). However, despite the expanded data-set, the overall picture of karyotype variability has changed significantly neither within the individual higher lineages nor within the Heteroptera in general. The lowest and the highest chromosome numbers remained the same, 2n=4 (*Lethocerus* sp., Belostomatidae, Nepomorpha) to 2n=80 (4 species of genus *Lopidea* Uhler 1872, Miridae, Cimicomorpha) as reported by Chickering (1927) and Akingbohungbe (1974), respectively; other species with these extreme counts were not found. The infraorder of semiaquatic bugs, Gerromorpha, in which the number of studied species has more than doubled (51 against 21), although enriched with new interesting data (Fairbairn 2016), can still be seen as a group only slightly varying in chromosome number, with predominantly XX/X(0) sex determination and the absence of m-chromosomes (as stated by Andersen 1982).

In recent years, knowledge of true bug cytogenetics has advanced significantly due to the use of modern techniques and approaches (chromosomal bandings, FISH, DNA content, etc.). For example, within the largest evolutionarily advanced and highly diversified infraorder Cimicomorpha, this applies to the families Cimicidae (e.g. Sadílek et al. 2019a, 2019a), Tingidae (Golub et al. 2015, 2016, 2017, 2018); Nabidae (Grozeva et al. 2004; Sadílek et al. 2020), and Reduviidae (e.g. Pita et al. 2014, 2016; Bardella et al. 2014; Grozeva et al. 2019). Specifically, Sadílek et al. (2020) using genome size data (together with rDNA-FISH) decided between two alternative hypotheses about the direction and mechanisms of karyotype evolution in the family Nabidae (Kuznetsova and Maryańska-Nadachowska 2000; Nokkala et al. 2007). The data obtained confirmed the hypothesis that the ancestral karyotype of Nabidae included 2n=16A+XY, and the karyotype 2n=32A+XY of *Himacerus* Wolff, 1811 spp. originated via polyploidization of autosomes (Kuznetsova et al. 2000). Then, Pita et al. (2016) and after them Grozeva et al. (2019) found out that the family Reduviidae, at least, the kissing bug subfamily Triatominae and the largest reduviid subfamily Harpactorinae, have the insect-type (TTAGG)*_n_* motif of telomeres. It should be noted that in true bugs this motif was first identified in *Lethoceruspatruelis* (Stål, 1854) from the family Belostomatidae, Nepomorpha (Kuznetsova et al. 2012), and all other true bug groups studied in this respect turned out to have lost the (TTAGG)*_n_* motif (Grozeva et al. 2011; Kuznetsova et al. 2012). In a series of publications on the family Tingidae (Golub et al. 2015, 2016, 2017, 2018) it was shown, firstly, that lace bugs also lost this telomere motif and, secondly, that closely related species of lace bugs share the same or similar karyotypes (at least, the same number of autosomes) but differ in the rDNA site location. Significant advances have been made recently in cytogenetics of the true water bug infraorder Nepomorpha, the families Nepidae, Aphelocheiridae and especially Belostomatidae, which is the best-studied family within this group in terms of karyotypes, meiosis and chromosome evolution (e.g., Kuznetsova et al. 2012; Chirino et al. 2013, 2017; Grozeva et al. 2013; Wisoram et al. 2013; Gallo et al. 2017). Species of the genus *Belostoma* Latreille 1807 (Belostomatidae) were shown to differ from one another in chromosome number and sex chromosome systems; besides, interstitial telomere sequences (ITS) found in some species were interpreted as signs of telomere-telomere fusions that took place in the evolution of the genus (Chirino et al. 2017). In the same family Belostomatidae, the species *Lethoceruspatruelis* (Stål, 1855) was found to have a conventional pre-reductional division of sex chromosomes in male meiosis, what distinguishes it from all other studied species of this family (Grozeva et al. 2013 and references therein). Although pre-reduction of sex chromosomes is not usual in Heteroptera, it does occur in some groups, and even closely related species occasionally differ in this pattern (Ueshima 1979; Grozeva et al. 2006, 2007). It shoud be noted in this regard that lace bugs (Tingidae, Cimicomorpha) are the only true bug family showing this meiotic pattern in all hitherto studied species (Ueshima 1979, Grozeva and Nokkala 2001; Golub et al. 2015, 2016, 2017, 2018). Recently, the first data on C-banding and FISH were published for the family Nepidae (Angus et al. 2017). At the same time, the first chromosomal data were obtained for the benthic true bug family Aphelocheiridae, in which all the three studied species, *Aphelocheirusaestivalis* (Fabricius, 1794), *A.murcius* Nieser et Millán, 1989, and *Aphelocheirus* sp. (from northern Spain), were shown to have the same karyotype, 2n=22+XX/X(0) (Stoianova et al. 2017).

### Coleorrhyncha

Coleorrhyncha (moss bugs or peloridiids) are little-known insects believed to be relict members of an ancient lineage of Hemiptera (Evans 1982). This taxonomically small group comprises 17 genera and 36 species of small insects (up to 5 mm long) with a cryptic lifestyle (Burckhardt 2009; Burckhardt et al. 2011). These “living fossils” inhabit temperate and sub-Antarctic rainforests of the southern Hemisphere, where they live in and feed on bryophytes and hepatics without moving much (Burckhardt 2009; Shcherbakov 2014). The phylogenetic relationships of moss bugs have been a matter of debates for a long time. In the past, Coleorrhyncha have been variously assigned to the Heteroptera or to the Homoptera since they possess a mixture of cicadomorphan and bug-like characters (Bechly and Szwedo 2007). Today, they are usually considered to be the sole family (the Peloridiidae) of the separate suborder Coleorrhyncha, which is treated as the sister group to the Heteroptera (Larivière et al. 2011), though there are data supporting divergent opinions as well (e.g. Cui et al. 2013). Recently, the first cytogenetic data on Coleorrhyncha were published. The species *Xenophyescascus* Bergroth, 1924 from New Zealand and *Peloridiumpomponorum* Shcherbakov, 2014 from Chile were reported to have 27(26A+X) and 33(2n=32A+X) holokinetic chromosomes, respectively (Grozeva et al. 2014; Kuznetsova et al. 2015). Besides, both species appeared to display the inverted sequence of sex chromosome divisions during spermatocyte meiosis, the so-called sex chromosome post-reduction previously known only in the Heteroptera (Ueshima 1979; Papeschi and Bressa 2006; Kuznetsova et al. 2011). This unique feature can be considered as an additional synapomorphy of Heteroptera + Coleorrhyncha (Kuznetsova et al. 2015; Wang et al. 2019).

## Conclusions

The overview presented here, shows that the supercohort Paraneoptera is a very diverse group, interesting for comparative cytogenetic studies, with different evolutionary scenarios from the maintenance of a preserved karyotype condition to greatly derived karyotype characteristics that can be traced within each of the higher-level taxa.

With only the intriguing exception of Thysanoptera, all Paraenoptera insects have holokinetic chromosomes. Paraneoptera have a great variety of sex chromosome systems, among which simple systems XX/XY and XX/X(0) clearly prevail. One or both systems are present in every major lineage, with rare exceptions where sex chromosomes either not identified or really absent (Thysanoptera, Parasita, and Aleyrodinea). The X(0) system has been recognized by different authors as the ancestral one for a number of groups including the most basal Copeognatha, and it appears to be an attractive candidate for the ancestral sex chromosome system for Paraneoptera clade in general (Fig. 1). Paraneoptera exhibit a large range of chromosome numbers varying from 2n=4 to 2n≈192. The lowest count is found in several groups including Parasita (*Gyropusovalis*, Amblicera, Mallophaga), HomopteraCoccinea (at least 5 species of the genus *Apiomorpha* from Eriococcidae, and at least 16 species in 6 genera of the tribe Iceryini, Margarodidae), HomopteraAphidinea (*Amphorophoratuberculata*, Aphididae), and Heteroptera (*Lethocerus* sp., Belostomatidae). On the other hand, the highest count is found in the only scale insect species, *Apiomorphamacqueeni*. The genera *Apiomorpha* (Coccinea) and *Amphorophora* (Aphidinea) are unique showing the most extensive chromosome number variability known among Coccinea and Aphidinea, respectively: in the first, 2n ranges from 4 to 192 (48-fold variation) and in the second, 2n ranges from 4 to 72 (13-fold variation). These examples suggest a high rate of chromosome evolution in *Apiomorpha* and *Amphorophora* and the potential for holokinetic chromosomes to break and fuse. It’s amazing that within *Apiomorpha*, as many as 42 chromosome counts have been reported for 47 species studied to date. It is important to note that both scale insects and aphids display very diverse and often very peculiar reproductive modes, including different types of parthenogenesis that may enable rearranged karyotypes to persist and potentially contribute to speciation events. However, all other Aphidococca as well as all other holokenetic groups of Paraneoptera display comparatively low chromosome numbers and rather little chromosome number variation at different taxonomic levels. The only other exception to this rule known today in Paraneoptera is the plant bug genus *Lopidea* (Miridae, Heteroptera) showing 2n=80 in each of the four studied species.

The currently available data suggest that the chromosome number variability in holokinetic groups of Paraneoptera is not very pronounced; it does not differ significantly from the variability observed in monocentric insects, including monocentric Thysanoptera (also classified within Paraneoptera) where 2n ranges from 20 to 100–106 (5-fold variation) in the only 17 studied species. It is worth mentioning in this regard that some other non-polyploid monocentric animals can also have high chromosome numbers as well as between-and within-species chromosome number variation (e.g. Contreras et al. 1990; Fetzner and Crandall 2001; Searle et al. 2019) including insects (e.g. Dutrillaux et al. 2007).

The significance of chromosomal rearrangements and mechanisms underlying differences in chromosome number have been debated for many years (e.g. White 1973, 1978; Grant 1981; King 1995; Schubert 2007). However, the question of how and why chromosome numbers evolve and why some groups have a wide variety of chromosome numbers, while others do not remains unanswered. As to holokinetic organisms, already in early classical publications, it was speculated that there is a certain mechanism preventing an increase in the number of chromosomes, and it happens quite rarely that spontaneous chromosome fragments are transmitted to subsequent generations and play a role in the evolution and speciation (Brown 1960; Nur et al. 1987).

As noted in the Introduction, Ruckman et al. (2020) conducted a purposeful study leading to the rather unexpected conclusion that rates of chromosome number evolution in holokinetic groups are similar to those in monocentric groups. We find that our analysis based on a large amount of data across the entire insect supercohort Paraneoptera supports the trends that have been seen by Ruckman et al. (2020). In our opinion, the hypothesis that rates of chromosome number evolution in holokinetic organisms are higher for the reason that they tolerate structural rearrangements of chromosomes better than monocentric organisms needs to be revised. We conclude that holokinetic chromosomes do have a well proven unique ability to fissions and fusions; however, these rearrangements only accidently, being probably influenced by certain environmental conditions, become drivers of evolutionary changes and speciation events, at least among Paraneoptera insects.
